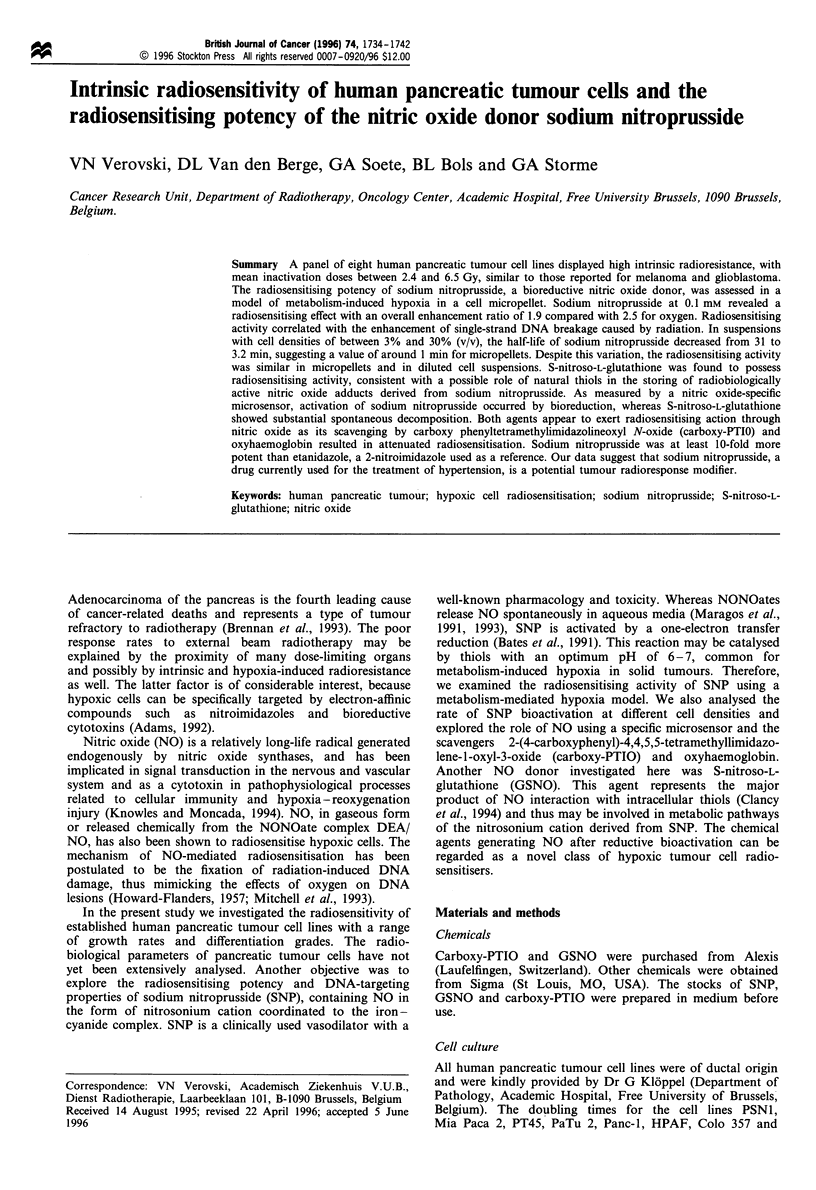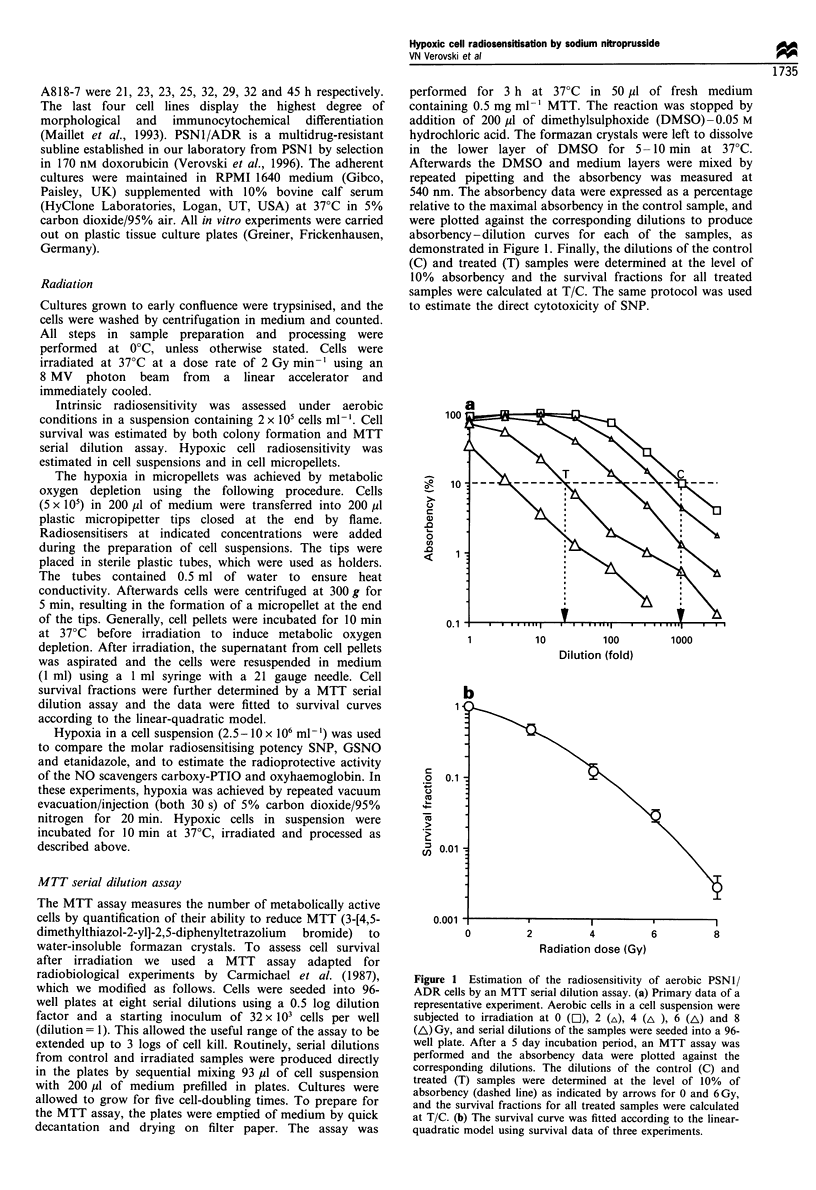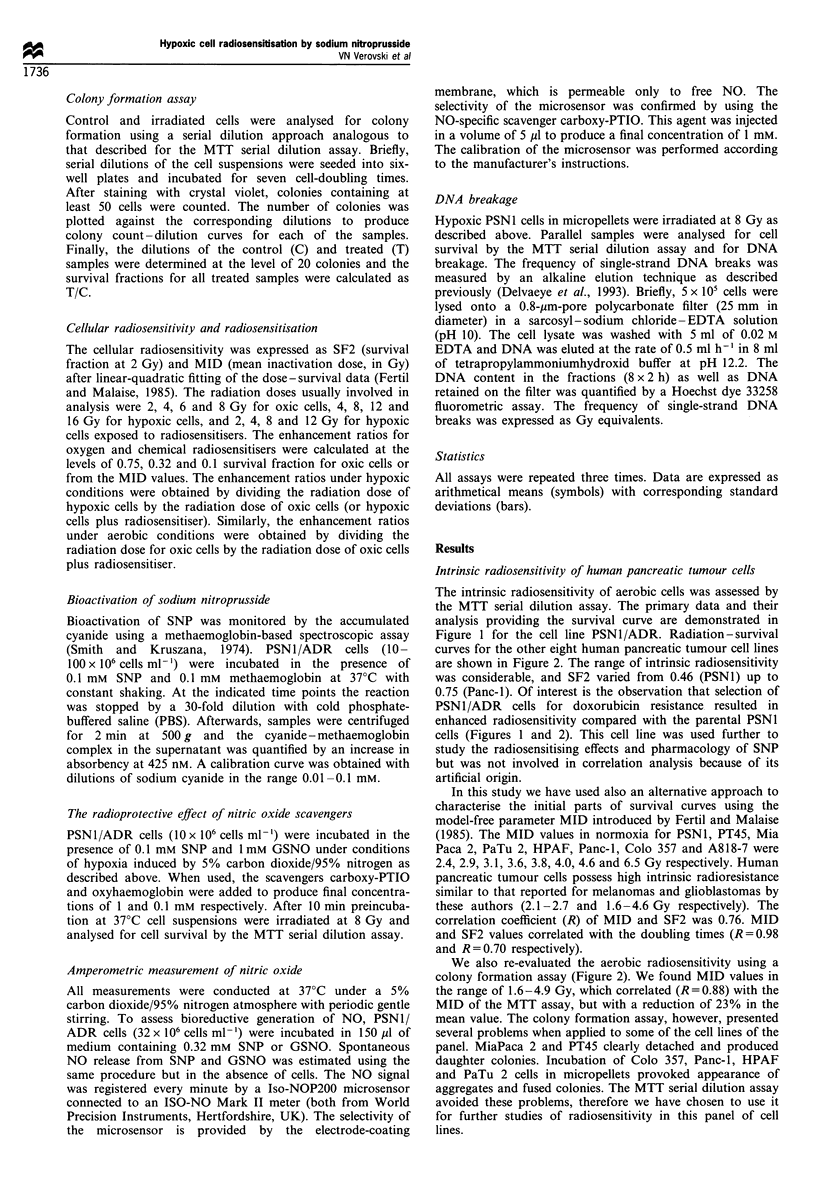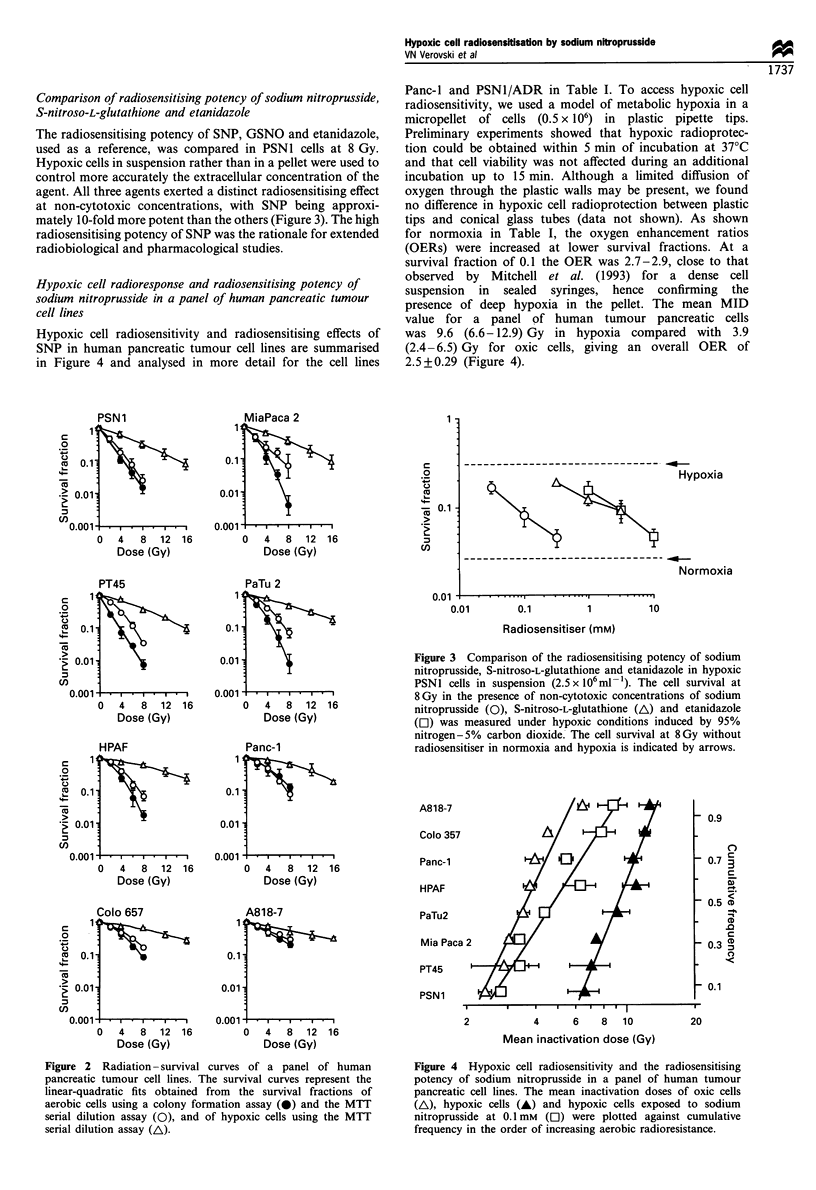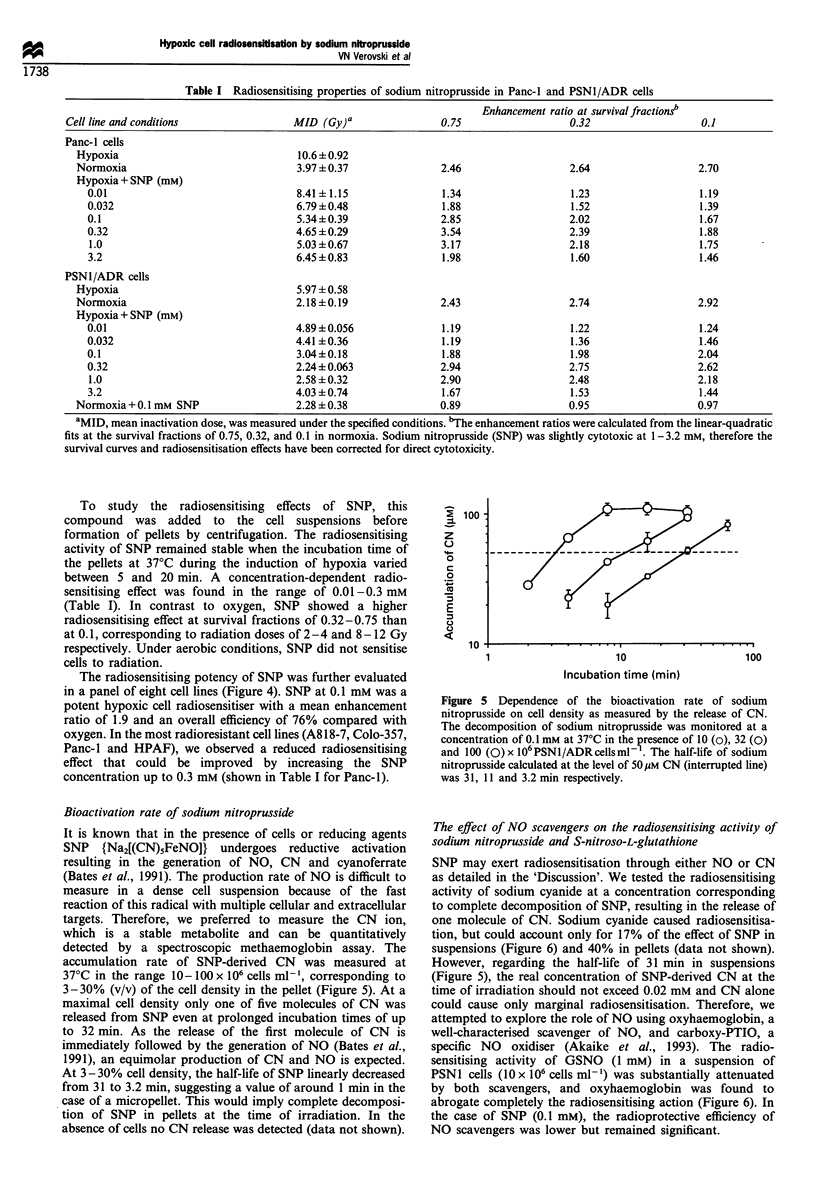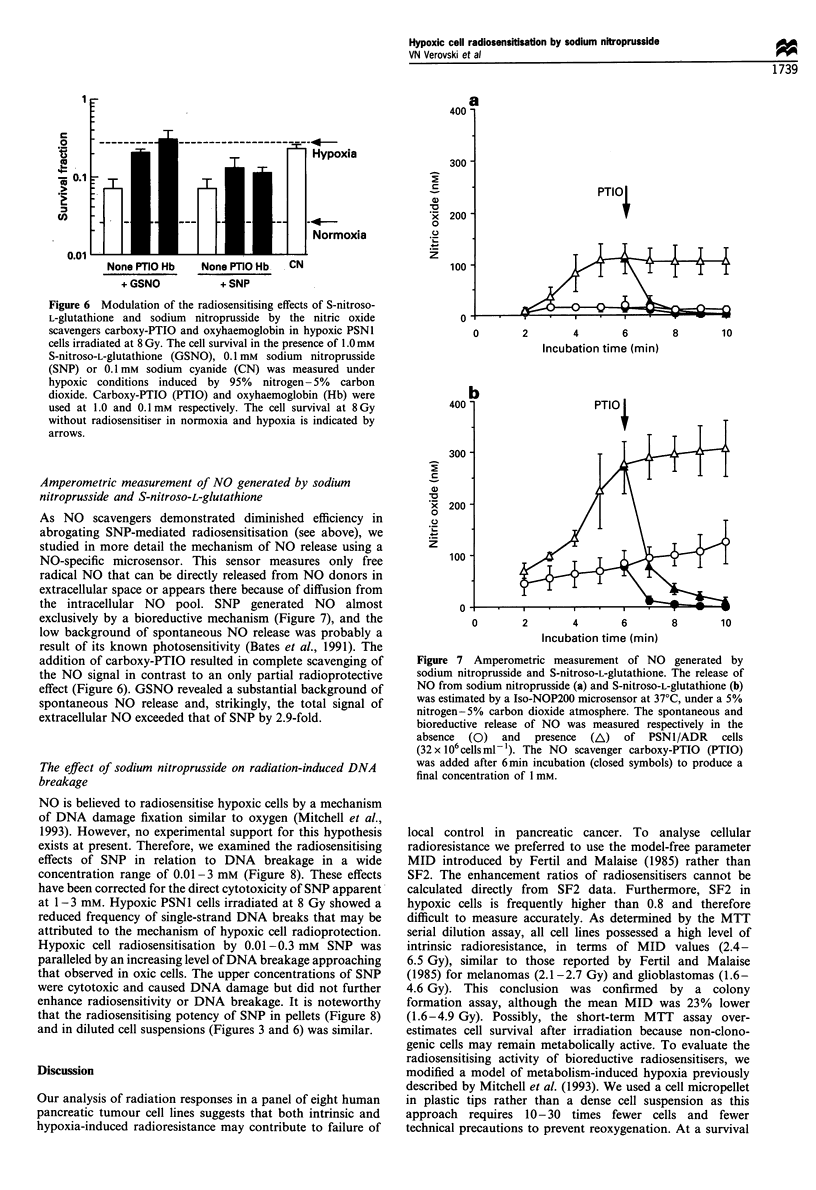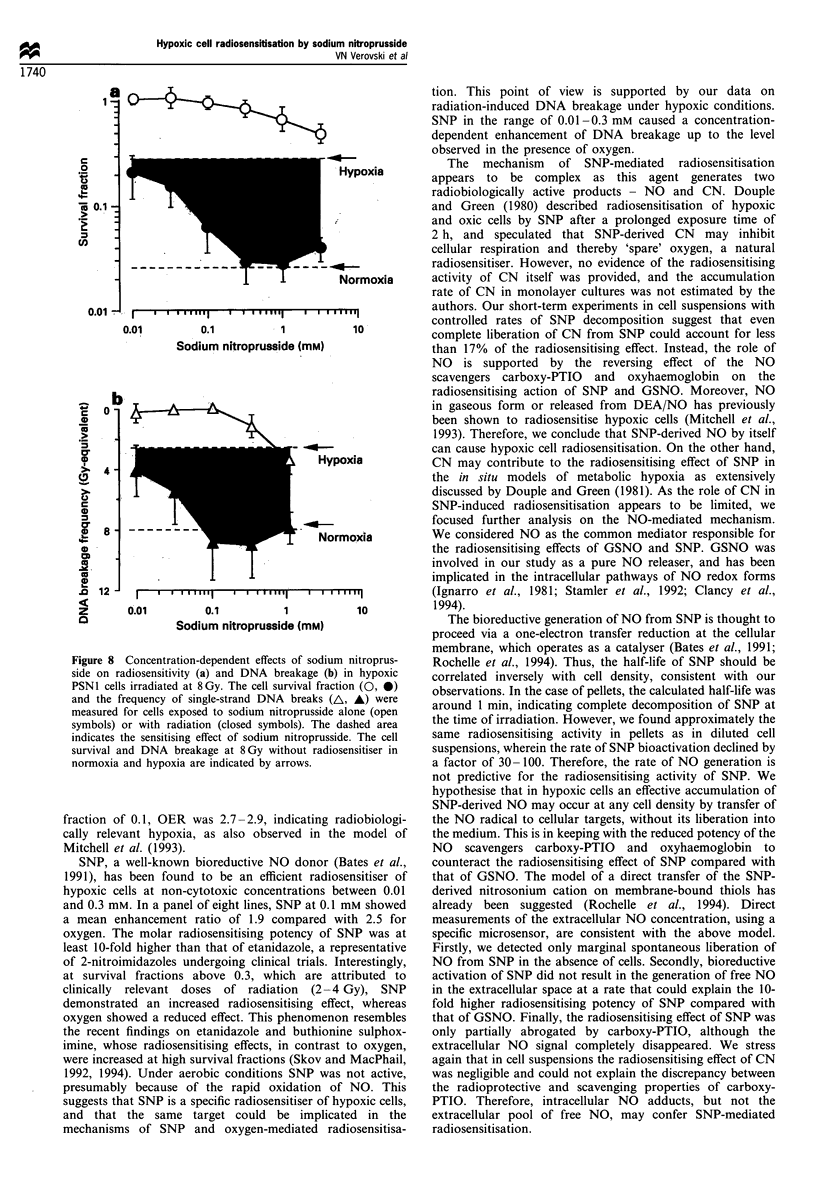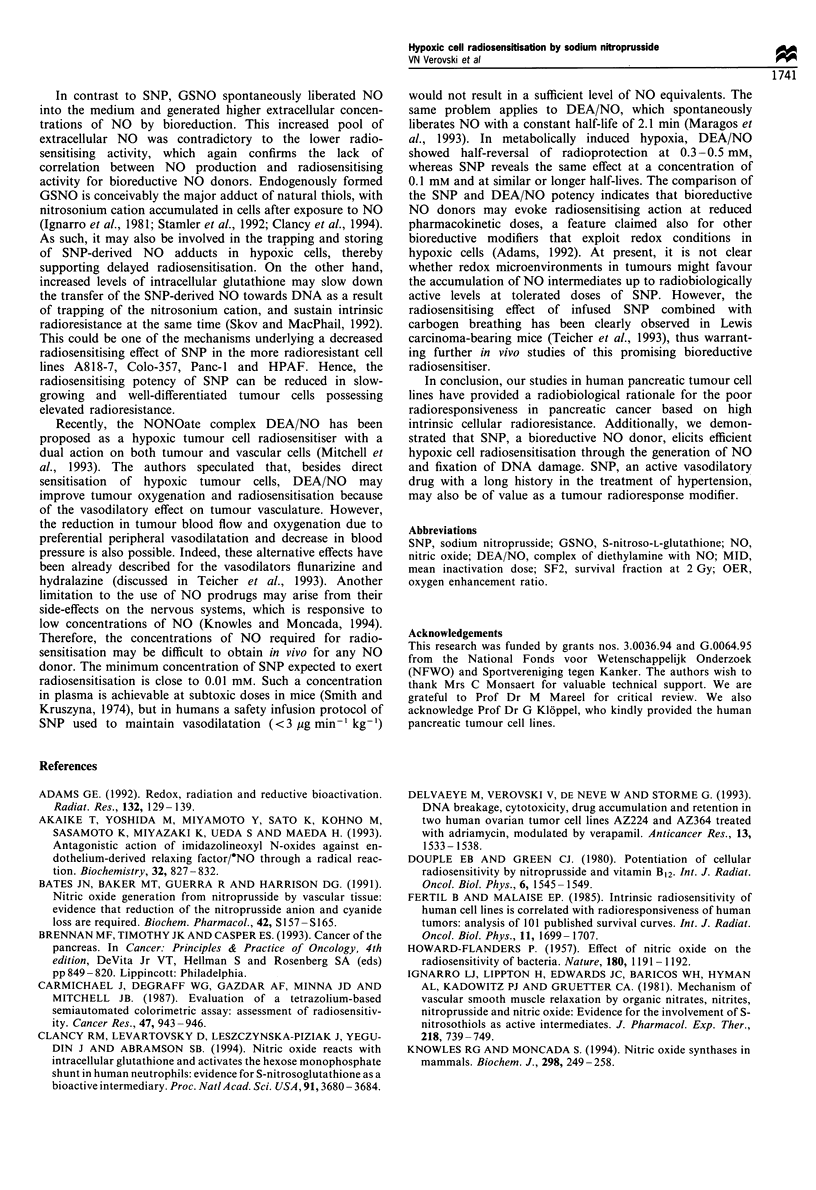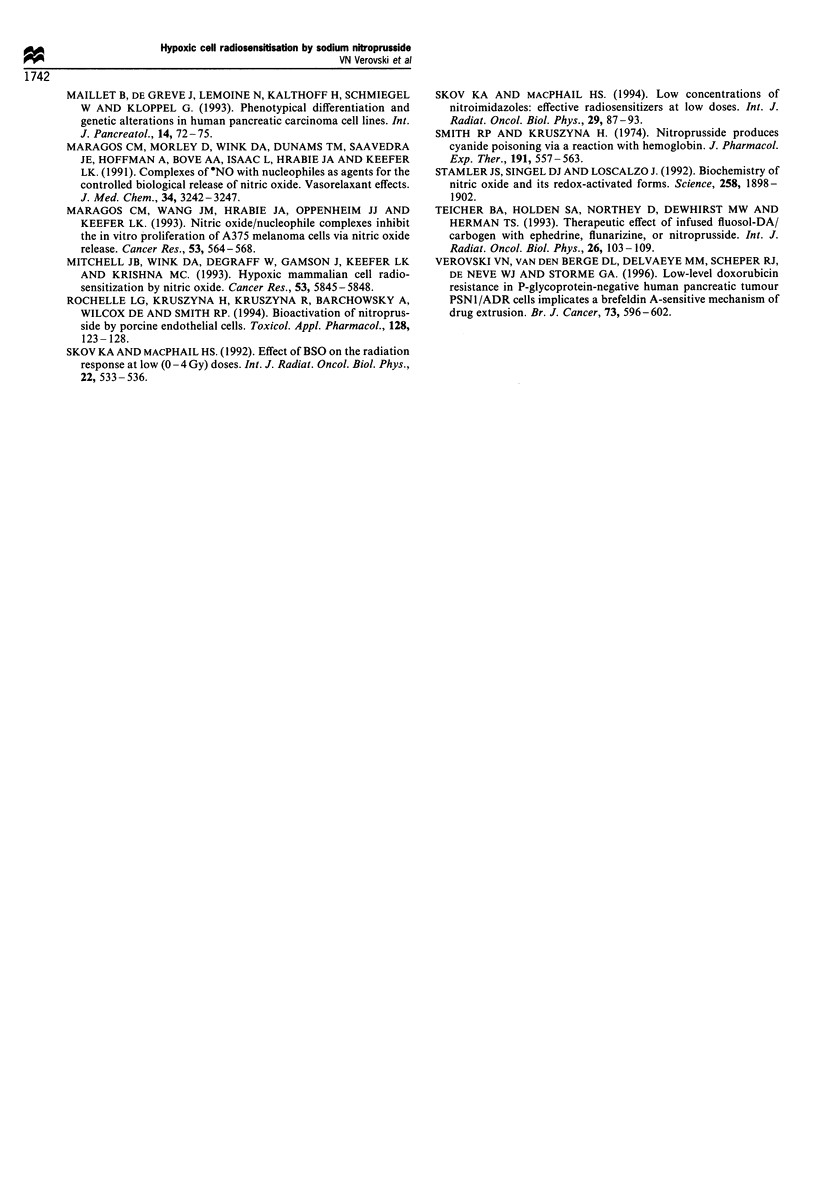# Intrinsic radiosensitivity of human pancreatic tumour cells and the radiosensitising potency of the nitric oxide donor sodium nitroprusside.

**DOI:** 10.1038/bjc.1996.623

**Published:** 1996-12

**Authors:** V. N. Verovski, D. L. Van den Berge, G. A. Soete, B. L. Bols, G. A. Storme

**Affiliations:** Department of Radiotherapy, Academic Hospital, Free University Brussels, Belgium.

## Abstract

A panel of eight human pancreatic tumour cell lines displayed high intrinsic radioresistance, with mean inactivation doses between 2.4 and 6.5 Gy, similar to those reported for melanoma and glioblastoma. The radiosensitising potency of sodium nitroprusside, a bioreductive nitric oxide donor, was assessed in a model of metabolism-induced hypoxia in a cell micropellet. Sodium nitroprusside at 0.1 mM revealed a radiosensitising effect with an overall enhancement ratio of 1.9 compared with 2.5 for oxygen. Radiosensitising activity correlated with the enhancement of single-strand DNA breakage caused by radiation. In suspensions with cell densities of between 3% and 30% (v/v), the half-life of sodium nitroprusside decreased from 31 to 3.2 min, suggesting a value of around 1 min for micropellets. Despite this variation, the radiosensitising activity was similar in micropellets and in diluted cell suspensions. S-nitroso-L-glutathione was found to possess radiosensitising activity, consistent with a possible role of natural thiols in the storing of radiobiologically active nitric oxide adducts derived from sodium nitroprusside. As measured by a nitric oxide-specific microsensor, activation of sodium nitroprusside occurred by bioreduction, whereas S-nitroso-L-glutathione showed substantial spontaneous decomposition. Both agents appear to exert radiosensitising action through nitric oxide as its scavenging by carboxy phenyltetramethylimidazolineoxyl N-oxide (carboxy-PTI0) and oxyhaemoglobin resulted in attenuated radiosensitisation. Sodium nitroprusside was at least 10-fold more potent than etanidazole, a 2-nitroimidazole used as a reference. Our data suggest that sodium nitroprusside, a drug currently used for the treatment of hypertension, is a potential tumour radioresponse modifier.


British Journal of Cancer (1996) 74, 1734-1742
© 1996 Stockton Press All rights reserved 0007-0920/96 $12.00

### Intrinsic radiosensitivity of human pancreatic tumour cells and the radiosensitising potency of the nitric oxide donor sodium nitroprusside

VN Verovski, DL Van den Berge, GA Soete, BL Bols and GA Storme

*Cancer Research Unit, Department of Radiotherapy, Oncology Center, Academic Hospital, Free University Brussels, 1090 Brussels, Belgium.*

**Summary** A panel of eight human pancreatic tumour cell lines displayed high intrinsic radioresistance, with mean inactivation doses between 2.4 and 6.5 Gy, similar to those reported for melanoma and glioblastoma. The radiosensitising potency of sodium nitroprusside, a bioreductive nitric oxide donor, was assessed in a model of metabolism-induced hypoxia in a cell micropellet. Sodium nitroprusside at 0.1 mM revealed a radiosensitising effect with an overall enhancement ratio of 1.9 compared with 2.5 for oxygen. Radiosensitising activity correlated with the enhancement of single-strand DNA breakage caused by radiation. In suspensions with cell densities of between 3% and 30% (v/v), the half-life of sodium nitroprusside decreased from 31 to 3.2 min, suggesting a value of around 1 min for micropellets. Despite this variation, the radiosensitising activity was similar in micropellets and in diluted cell suspensions. S-nitroso-L-glutathione was found to possess radiosensitising activity, consistent with a possible role of natural thiols in the storing of radiobiologically active nitric oxide adducts derived from sodium nitroprusside. As measured by a nitric oxide-specific microsensor, activation of sodium nitroprusside occurred by bioreduction, whereas S-nitroso-L-glutathione showed substantial spontaneous decomposition. Both agents appear to exert radiosensitising action through nitric oxide as its scavenging by carboxy phenyltetramethylimidazolineoxyl *N*-oxide (carboxy-PTI0) and oxyhaemoglobin resulted in attenuated radiosensitisation. Sodium nitroprusside was at least 10-fold more potent than etanidazole, a 2-nitroimidazole used as a reference. Our data suggest that sodium nitroprusside, a drug currently used for the treatment of hypertension, is a potential tumour radioresponse modifier.

**Keywords:** human pancreatic tumour; hypoxic cell radiosensitisation; sodium nitroprusside; S-nitroso-L-glutathione; nitric oxide

Adenocarcinoma of the pancreas is the fourth leading cause of cancer-related deaths and represents a type of tumour refractory to radiotherapy (Brennan *et al.*, 1993). The poor response rates to external beam radiotherapy may be explained by the proximity of many dose-limiting organs and possibly by intrinsic and hypoxia-induced radioresistance as well. The latter factor is of considerable interest, because hypoxic cells can be specifically targeted by electron-affinic compounds such as nitroimidazoles and bioreductive cytotoxins (Adams, 1992).

Nitric oxide (NO) is a relatively long-life radical generated endogenously by nitric oxide synthases, and has been implicated in signal transduction in the nervous and vascular system and as a cytotoxin in pathophysiological processes related to cellular immunity and hypoxia-reoxygenation injury (Knowles and Moncada, 1994). NO, in gaseous form or released chemically from the NONOate complex DEA/NO, has also been shown to radiosensitise hypoxic cells. The mechanism of NO-mediated radiosensitisation has been postulated to be the fixation of radiation-induced DNA damage, thus mimicking the effects of oxygen on DNA lesions (Howard-Flanders, 1957; Mitchell *et al.*, 1993).

In the present study we investigated the radiosensitivity of established human pancreatic tumour cell lines with a range of growth rates and differentiation grades. The radiobiological parameters of pancreatic tumour cells have not yet been extensively analysed. Another objective was to explore the radiosensitising potency and DNA-targeting properties of sodium nitroprusside (SNP), containing NO in the form of nitrosonium cation coordinated to the iron-cyanide complex. SNP is a clinically used vasodilator with a well-known pharmacology and toxicity. Whereas NONOates release NO spontaneously in aqueous media (Maragos *et al.*, 1991, 1993), SNP is activated by a one-electron transfer reduction (Bates *et al.*, 1991). This reaction may be catalysed by thiols with an optimum pH of 6-7, common for metabolism-induced hypoxia in solid tumours. Therefore, we examined the radiosensitising activity of SNP using a metabolism-mediated hypoxia model. We also analysed the rate of SNP bioactivation at different cell densities and explored the role of NO using a specific microsensor and the scavengers 2-(4-carboxyphenyl)-4,4,5,5-tetramethyllimidazolene-1-oxyl-3-oxide (carboxy-PTIO) and oxyhaemoglobin. Another NO donor investigated here was S-nitroso-L-glutathione (GSNO). This agent represents the major product of NO interaction with intracellular thiols (Clancy *et al.*, 1994) and thus may be involved in metabolic pathways of the nitrosonium cation derived from SNP. The chemical agents generating NO after reductive bioactivation can be regarded as a novel class of hypoxic tumour cell radiosensitisers.

Correspondence: VN Verovski, Academisch Ziekenhuis V.U.B., Dienst Radiotherapie, Laarbeeklaan 101, B-1090 Brussels, Belgium
Received 14 August 1995; revised 22 April 1996; accepted 5 June 1996

### Materials and methods

### *Chemicals*

Carboxy-PTIO and GSNO were purchased from Alexis (Laufelfingen, Switzerland). Other chemicals were obtained from Sigma (St Louis, MO, USA). The stocks of SNP, GSNO and carboxy-PTIO were prepared in medium before use.

### *Cell culture*

All human pancreatic tumour cell lines were of ductal origin and were kindly provided by Dr G Klöppel (Department of Pathology, Academic Hospital, Free University of Brussels, Belgium). The doubling times for the cell lines PSN1, Mia Paca 2, PT45, PaTu 2, Panc-1, HPAF, Colo 357 and

A818-7 were 21, 23, 23, 25, 32, 29, 32 and 45 h respectively. The last four cell lines display the highest degree of morphological and immunocytochemical differentiation (Maillet *et al.*, 1993). PSN1/ADR is a multidrug-resistant subline established in our laboratory from PSN1 by selection in 170 nM doxorubicin (Verovski *et al.*, 1996). The adherent cultures were maintained in RPMI 1640 medium (Gibco, Paisley, UK) supplemented with 10% bovine calf serum (HyClone Laboratories, Logan, UT, USA) at 37°C in 5% carbon dioxide/95% air. All *in vitro* experiments were carried out on plastic tissue culture plates (Greiner, Frickenhausen, Germany).

### *Radiation*

Cultures grown to early confluence were trypsinised, and the cells were washed by centrifugation in medium and counted. All steps in sample preparation and processing were performed at 0°C, unless otherwise stated. Cells were irradiated at 37°C at a dose rate of 2 Gy $min^{-1}$ using an 8 MV photon beam from a linear accelerator and immediately cooled.

Intrinsic radiosensitivity was assessed under aerobic conditions in a suspension containing $2 \times 10^5$ cells $ml^{-1}$. Cell survival was estimated by both colony formation and MTT serial dilution assay. Hypoxic cell radiosensitivity was estimated in cell suspensions and in cell micropellets.

The hypoxia in micropellets was achieved by metabolic oxygen depletion using the following procedure. Cells ($5 \times 10^5$) in 200 $\mu$l of medium were transferred into 200 $\mu$l plastic micropipetter tips closed at the end by flame. Radiosensitisers at indicated concentrations were added during the preparation of cell suspensions. The tips were placed in sterile plastic tubes, which were used as holders. The tubes contained 0.5 ml of water to ensure heat conductivity. Afterwards cells were centrifuged at 300 ***g*** for 5 min, resulting in the formation of a micropellet at the end of the tips. Generally, cell pellets were incubated for 10 min at 37°C before irradiation to induce metabolic oxygen depletion. After irradiation, the supernatant from cell pellets was aspirated and the cells were resuspended in medium (1 ml) using a 1 ml syringe with a 21 gauge needle. Cell survival fractions were further determined by a MTT serial dilution assay and the data were fitted to survival curves according to the linear-quadratic model.

Hypoxia in a cell suspension ($2.5-10 \times 10^6$ $ml^{-1}$) was used to compare the molar radiosensitising potency SNP, GSNO and etanidazole, and to estimate the radioprotective activity of the NO scavengers carboxy-PTIO and oxyhaemoglobin. In these experiments, hypoxia was achieved by repeated vacuum evacuation/injection (both 30 s) of 5% carbon dioxide/95% nitrogen for 20 min. Hypoxic cells in suspension were incubated for 10 min at 37°C, irradiated and processed as described above.

### *MTT serial dilution assay*

The MTT assay measures the number of metabolically active cells by quantification of their ability to reduce MTT (3-[4,5-dimethylthiazol-2-yl]-2,5-diphenyltetrazolium bromide) to water-insoluble formazan crystals. To assess cell survival after irradiation we used a MTT assay adapted for radiobiological experiments by Carmichael *et al.* (1987), which we modified as follows. Cells were seeded into 96-well plates at eight serial dilutions using a 0.5 log dilution factor and a starting inoculum of $32 \times 10^3$ cells per well (dilution = 1). This allowed the useful range of the assay to be extended up to 3 logs of cell kill. Routinely, serial dilutions from control and irradiated samples were produced directly in the plates by sequential mixing 93 $\mu$l of cell suspension with 200 $\mu$l of medium prefilled in plates. Cultures were allowed to grow for five cell-doubling times. To prepare for the MTT assay, the plates were emptied of medium by quick decantation and drying on filter paper. The assay was performed for 3 h at 37°C in 50 $\mu$l of fresh medium containing 0.5 mg $ml^{-1}$ MTT. The reaction was stopped by addition of 200 $\mu$l of dimethylsulphoxide (DMSO)–0.05 M hydrochloric acid. The formazan crystals were left to dissolve in the lower layer of DMSO for 5–10 min at 37°C. Afterwards the DMSO and medium layers were mixed by repeated pipetting and the absorbency was measured at 540 nm. The absorbency data were expressed as a percentage relative to the maximal absorbency in the control sample, and were plotted against the corresponding dilutions to produce absorbency–dilution curves for each of the samples, as demonstrated in Figure 1. Finally, the dilutions of the control (C) and treated (T) samples were determined at the level of 10% absorbency and the survival fractions for all treated samples were calculated at T/C. The same protocol was used to estimate the direct cytotoxicity of SNP.

**Figure 1** Estimation of the radiosensitivity of aerobic PSN1/ADR cells by an MTT serial dilution assay. (**a**) Primary data of a representative experiment. Aerobic cells in a cell suspension were subjected to irradiation at 0 (□), 2 (△), 4 (△), 6 (△) and 8 (△) Gy, and serial dilutions of the samples were seeded into a 96-well plate. After a 5 day incubation period, an MTT assay was performed and the absorbency data were plotted against the corresponding dilutions. The dilutions of the control (C) and treated (T) samples were determined at the level of 10% of absorbency (dashed line) as indicated by arrows for 0 and 6 Gy, and the survival fractions for all treated samples were calculated at T/C. (**b**) The survival curve was fitted according to the linear-quadratic model using survival data of three experiments.

*Colony formation assay*

Control and irradiated cells were analysed for colony formation using a serial dilution approach analogous to that described for the MTT serial dilution assay. Briefly, serial dilutions of the cell suspensions were seeded into six-well plates and incubated for seven cell-doubling times. After staining with crystal violet, colonies containing at least 50 cells were counted. The number of colonies was plotted against the corresponding dilutions to produce colony count–dilution curves for each of the samples. Finally, the dilutions of the control (C) and treated (T) samples were determined at the level of 20 colonies and the survival fractions for all treated samples were calculated as T/C.

*Cellular radiosensitivity and radiosensitisation*

The cellular radiosensitivity was expressed as SF2 (survival fraction at 2 Gy) and MID (mean inactivation dose, in Gy) after linear-quadratic fitting of the dose–survival data (Fertil and Malaise, 1985). The radiation doses usually involved in analysis were 2, 4, 6 and 8 Gy for oxic cells, 4, 8, 12 and 16 Gy for hypoxic cells, and 2, 4, 8 and 12 Gy for hypoxic cells exposed to radiosensitisers. The enhancement ratios for oxygen and chemical radiosensitisers were calculated at the levels of 0.75, 0.32 and 0.1 survival fraction for oxic cells or from the MID values. The enhancement ratios under hypoxic conditions were obtained by dividing the radiation dose of hypoxic cells by the radiation dose of oxic cells (or hypoxic cells plus radiosensitiser). Similarly, the enhancement ratios under aerobic conditions were obtained by dividing the radiation dose for oxic cells by the radiation dose of oxic cells plus radiosensitiser.

*Bioactivation of sodium nitroprusside*

Bioactivation of SNP was monitored by the accumulated cyanide using a methaemoglobin-based spectroscopic assay (Smith and Kruszana, 1974). PSN1/ADR cells ($10-100\times10^6$ cells $ml^{-1}$) were incubated in the presence of 0.1 mM SNP and 0.1 mM methaemoglobin at 37°C with constant shaking. At the indicated time points the reaction was stopped by a 30-fold dilution with cold phosphate-buffered saline (PBS). Afterwards, samples were centrifuged for 2 min at 500 *g* and the cyanide–methaemoglobin complex in the supernatant was quantified by an increase in absorbency at 425 nM. A calibration curve was obtained with dilutions of sodium cyanide in the range 0.01–0.1 mM.

*The radioprotective effect of nitric oxide scavengers*

PSN1/ADR cells ($10\times10^6$ cells $ml^{-1}$) were incubated in the presence of 0.1 mM SNP and 1 mM GSNO under conditions of hypoxia induced by 5% carbon dioxide/95% nitrogen as described above. When used, the scavengers carboxy-PTIO and oxyhaemoglobin were added to produce final concentrations of 1 and 0.1 mM respectively. After 10 min preincubation at 37°C cell suspensions were irradiated at 8 Gy and analysed for cell survival by the MTT serial dilution assay.

*Amperometric measurement of nitric oxide*

All measurements were conducted at 37°C under a 5% carbon dioxide/95% nitrogen atmosphere with periodic gentle stirring. To assess bioreductive generation of NO, PSN1/ADR cells ($32\times10^6$ cells $ml^{-1}$) were incubated in 150 $\mu$l of medium containing 0.32 mM SNP or GSNO. Spontaneous NO release from SNP and GSNO was estimated using the same procedure but in the absence of cells. The NO signal was registered every minute by a Iso-NOP200 microsensor connected to an ISO-NO Mark II meter (both from World Precision Instruments, Hertfordshire, UK). The selectivity of the microsensor is provided by the electrode-coating membrane, which is permeable only to free NO. The selectivity of the microsensor was confirmed by using the NO-specific scavenger carboxy-PTIO. This agent was injected in a volume of 5 $\mu$l to produce a final concentration of 1 mM. The calibration of the microsensor was performed according to the manufacturer's instructions.

*DNA breakage*

Hypoxic PSN1 cells in micropellets were irradiated at 8 Gy as described above. Parallel samples were analysed for cell survival by the MTT serial dilution assay and for DNA breakage. The frequency of single-strand DNA breaks was measured by an alkaline elution technique as described previously (Delvaeye *et al.*, 1993). Briefly, $5\times10^5$ cells were lysed onto a 0.8-$\mu$m-pore polycarbonate filter (25 mm in diameter) in a sarcosyl–sodium chloride–EDTA solution (pH 10). The cell lysate was washed with 5 ml of 0.02 M EDTA and DNA was eluted at the rate of 0.5 ml $h^{-1}$ in 8 ml of tetrapropylammoniumhydroxid buffer at pH 12.2. The DNA content in the fractions ($8\times2$ h) as well as DNA retained on the filter was quantified by a Hoechst dye 33258 fluorometric assay. The frequency of single-strand DNA breaks was expressed as Gy equivalents.

*Statistics*

All assays were repeated three times. Data are expressed as arithmetical means (symbols) with corresponding standard deviations (bars).

### Results

*Intrinsic radiosensitivity of human pancreatic tumour cells*

The intrinsic radiosensitivity of aerobic cells was assessed by the MTT serial dilution assay. The primary data and their analysis providing the survival curve are demonstrated in Figure 1 for the cell line PSN1/ADR. Radiation–survival curves for the other eight human pancreatic tumour cell lines are shown in Figure 2. The range of intrinsic radiosensitivity was considerable, and SF2 varied from 0.46 (PSN1) up to 0.75 (Panc-1). Of interest is the observation that selection of PSN1/ADR cells for doxorubicin resistance resulted in enhanced radiosensitivity compared with the parental PSN1 cells (Figures 1 and 2). This cell line was used further to study the radiosensitising effects and pharmacology of SNP but was not involved in correlation analysis because of its artificial origin.

In this study we have used also an alternative approach to characterise the initial parts of survival curves using the model-free parameter MID introduced by Fertil and Malaise (1985). The MID values in normoxia for PSN1, PT45, Mia Paca 2, PaTu 2, HPAF, Panc-1, Colo 357 and A818-7 were 2.4, 2.9, 3.1, 3.6, 3.8, 4.0, 4.6 and 6.5 Gy respectively. Human pancreatic tumour cells possess high intrinsic radioresistance similar to that reported for melanomas and glioblastomas by these authors (2.1–2.7 and 1.6–4.6 Gy respectively). The correlation coefficient ($R$) of MID and SF2 was 0.76. MID and SF2 values correlated with the doubling times ($R=0.98$ and $R=0.70$ respectively).

We also re-evaluated the aerobic radiosensitivity using a colony formation assay (Figure 2). We found MID values in the range of 1.6–4.9 Gy, which correlated ($R=0.88$) with the MID of the MTT assay, but with a reduction of 23% in the mean value. The colony formation assay, however, presented several problems when applied to some of the cell lines of the panel. MiaPaca 2 and PT45 clearly detached and produced daughter colonies. Incubation of Colo 357, Panc-1, HPAF and PaTu 2 cells in micropellets provoked appearance of aggregates and fused colonies. The MTT serial dilution assay avoided these problems, therefore we have chosen to use it for further studies of radiosensitivity in this panel of cell lines.

*Comparison of radiosensitising potency of sodium nitroprusside, S-nitroso-L-glutathione and etanidazole*

The radiosensitising potency of SNP, GSNO and etanidazole, used as a reference, was compared in PSN1 cells at 8 Gy. Hypoxic cells in suspension rather than in a pellet were used to control more accurately the extracellular concentration of the agent. All three agents exerted a distinct radiosensitising effect at non-cytotoxic concentrations, with SNP being approximately 10-fold more potent than the others (Figure 3). The high radiosensitising potency of SNP was the rationale for extended radiobiological and pharmacological studies.

*Hypoxic cell radioresponse and radiosensitising potency of sodium nitroprusside in a panel of human pancreatic tumour cell lines*

Hypoxic cell radiosensitivity and radiosensitising effects of SNP in human pancreatic tumour cell lines are summarised in Figure 4 and analysed in more detail for the cell lines Panc-1 and PSN1/ADR in Table I. To access hypoxic cell radiosensitivity, we used a model of metabolic hypoxia in a micropellet of cells ($0.5 \times 10^6$) in plastic pipette tips. Preliminary experiments showed that hypoxic radioprotection could be obtained within 5 min of incubation at 37°C and that cell viability was not affected during an additional incubation up to 15 min. Although a limited diffusion of oxygen through the plastic walls may be present, we found no difference in hypoxic cell radioprotection between plastic tips and conical glass tubes (data not shown). As shown for normoxia in Table I, the oxygen enhancement ratios (OERs) were increased at lower survival fractions. At a survival fraction of 0.1 the OER was 2.7–2.9, close to that observed by Mitchell *et al.* (1993) for a dense cell suspension in sealed syringes, hence confirming the presence of deep hypoxia in the pellet. The mean MID value for a panel of human tumour pancreatic cells was 9.6 (6.6–12.9) Gy in hypoxia compared with 3.9 (2.4–6.5) Gy for oxic cells, giving an overall OER of $2.5 \pm 0.29$ (Figure 4).

**Figure 2** Radiation–survival curves of a panel of human pancreatic tumour cell lines. The survival curves represent the linear-quadratic fits obtained from the survival fractions of aerobic cells using a colony formation assay (●) and the MTT serial dilution assay (○), and of hypoxic cells using the MTT serial dilution assay (△).

**Figure 3** Comparison of the radiosensitising potency of sodium nitroprusside, S-nitroso-L-glutathione and etanidazole in hypoxic PSN1 cells in suspension ($2.5 \times 10^6 ml^{-1}$). The cell survival at 8 Gy in the presence of non-cytotoxic concentrations of sodium nitroprusside (○), S-nitroso-L-glutathione (△) and etanidazole (□) was measured under hypoxic conditions induced by 95% nitrogen–5% carbon dioxide. The cell survival at 8 Gy without radiosensitiser in normoxia and hypoxia is indicated by arrows.

**Figure 4** Hypoxic cell radiosensitivity and the radiosensitising potency of sodium nitroprusside in a panel of human tumour pancreatic cell lines. The mean inactivation doses of oxic cells (△), hypoxic cells (▲) and hypoxic cells exposed to sodium nitroprusside at 0.1 mM (□) were plotted against cumulative frequency in the order of increasing aerobic radioresistance.

**Table I** Radiosensitising properties of sodium nitroprusside in Panc-1 and PSN1/ADR cells

| Cell line and conditions | MID (Gy)[a] | Enhancement ratio at survival fractions[b] 0.75 | 0.32 | 0.1 |
|---|---|---|---|---|
| Panc-1 cells | | | | |
| Hypoxia | 10.6±0.92 | | | |
| Normoxia | 3.97±0.37 | 2.46 | 2.64 | 2.70 |
| Hypoxia+SNP (mM) | | | | |
| 0.01 | 8.41±1.15 | 1.34 | 1.23 | 1.19 |
| 0.032 | 6.79±0.48 | 1.88 | 1.52 | 1.39 |
| 0.1 | 5.34±0.39 | 2.85 | 2.02 | 1.67 |
| 0.32 | 4.65±0.29 | 3.54 | 2.39 | 1.88 |
| 1.0 | 5.03±0.67 | 3.17 | 2.18 | 1.75 |
| 3.2 | 6.45±0.83 | 1.98 | 1.60 | 1.46 |
| PSN1/ADR cells | | | | |
| Hypoxia | 5.97±0.58 | | | |
| Normoxia | 2.18±0.19 | 2.43 | 2.74 | 2.92 |
| Hypoxia+SNP (mM) | | | | |
| 0.01 | 4.89±0.056 | 1.19 | 1.22 | 1.24 |
| 0.032 | 4.41±0.36 | 1.19 | 1.36 | 1.46 |
| 0.1 | 3.04±0.18 | 1.88 | 1.98 | 2.04 |
| 0.32 | 2.24±0.063 | 2.94 | 2.75 | 2.62 |
| 1.0 | 2.58±0.32 | 2.90 | 2.48 | 2.18 |
| 3.2 | 4.03±0.74 | 1.67 | 1.53 | 1.44 |
| Normoxia+0.1 mM SNP | 2.28±0.38 | 0.89 | 0.95 | 0.97 |

[a]MID, mean inactivation dose, was measured under the specified conditions. [b]The enhancement ratios were calculated from the linear-quadratic fits at the survival fractions of 0.75, 0.32, and 0.1 in normoxia. Sodium nitroprusside (SNP) was slightly cytotoxic at 1–3.2 mM, therefore the survival curves and radiosensitisation effects have been corrected for direct cytotoxicity.

To study the radiosensitising effects of SNP, this compound was added to the cell suspensions before formation of pellets by centrifugation. The radiosensitising activity of SNP remained stable when the incubation time of the pellets at 37°C during the induction of hypoxia varied between 5 and 20 min. A concentration-dependent radiosensitising effect was found in the range of 0.01–0.3 mM (Table I). In contrast to oxygen, SNP showed a higher radiosensitising effect at survival fractions of 0.32–0.75 than at 0.1, corresponding to radiation doses of 2–4 and 8–12 Gy respectively. Under aerobic conditions, SNP did not sensitise cells to radiation.

The radiosensitising potency of SNP was further evaluated in a panel of eight cell lines (Figure 4). SNP at 0.1 mM was a potent hypoxic cell radiosensitiser with a mean enhancement ratio of 1.9 and an overall efficiency of 76% compared with oxygen. In the most radioresistant cell lines (A818-7, Colo-357, Panc-1 and HPAF), we observed a reduced radiosensitising effect that could be improved by increasing the SNP concentration up to 0.3 mM (shown in Table I for Panc-1).

**Figure 5** Dependence of the bioactivation rate of sodium nitroprusside on cell density as measured by the release of CN. The decomposition of sodium nitroprusside was monitored at a concentration of 0.1 mM at 37°C in the presence of 10 (o), 32 (O) and 100 (O) × $10^6$ PSN1/ADR cells ml$^{-1}$. The half-life of sodium nitroprusside calculated at the level of 50 µM CN (interrupted line) was 31, 11 and 3.2 min respectively.

*Bioactivation rate of sodium nitroprusside*

It is known that in the presence of cells or reducing agents SNP {$Na_2[(CN)_5FeNO]$} undergoes reductive activation resulting in the generation of NO, CN and cyanoferrate (Bates *et al.*, 1991). The production rate of NO is difficult to measure in a dense cell suspension because of the fast reaction of this radical with multiple cellular and extracellular targets. Therefore, we preferred to measure the CN ion, which is a stable metabolite and can be quantitatively detected by a spectroscopic methaemoglobin assay. The accumulation rate of SNP-derived CN was measured at 37°C in the range 10–100 × $10^6$ cells ml$^{-1}$, corresponding to 3–30% (v/v) of the cell density in the pellet (Figure 5). At a maximal cell density only one of five molecules of CN was released from SNP even at prolonged incubation times of up to 32 min. As the release of the first molecule of CN is immediately followed by the generation of NO (Bates *et al.*, 1991), an equimolar production of CN and NO is expected. At 3–30% cell density, the half-life of SNP linearly decreased from 31 to 3.2 min, suggesting a value of around 1 min in the case of a micropellet. This would imply complete decomposition of SNP in pellets at the time of irradiation. In the absence of cells no CN release was detected (data not shown).

*The effect of NO scavengers on the radiosensitising activity of sodium nitroprusside and S-nitroso-L-glutathione*

SNP may exert radiosensitisation through either NO or CN as detailed in the 'Discussion'. We tested the radiosensitising activity of sodium cyanide at a concentration corresponding to complete decomposition of SNP, resulting in the release of one molecule of CN. Sodium cyanide caused radiosensitisation, but could account only for 17% of the effect of SNP in suspensions (Figure 6) and 40% in pellets (data not shown). However, regarding the half-life of 31 min in suspensions (Figure 5), the real concentration of SNP-derived CN at the time of irradiation should not exceed 0.02 mM and CN alone could cause only marginal radiosensitisation. Therefore, we attempted to explore the role of NO using oxyhaemoglobin, a well-characterised scavenger of NO, and carboxy-PTIO, a specific NO oxidiser (Akaike *et al.*, 1993). The radiosensitising activity of GSNO (1 mM) in a suspension of PSN1 cells (10 × $10^6$ cells ml$^{-1}$) was substantially attenuated by both scavengers, and oxyhaemoglobin was found to abrogate completely the radiosensitising action (Figure 6). In the case of SNP (0.1 mM), the radioprotective efficiency of NO scavengers was lower but remained significant.

**Figure 6** Modulation of the radiosensitising effects of S-nitroso-L-glutathione and sodium nitroprusside by the nitric oxide scavengers carboxy-PTIO and oxyhaemoglobin in hypoxic PSN1 cells irradiated at 8 Gy. The cell survival in the presence of 1.0 mM S-nitroso-L-glutathione (GSNO), 0.1 mM sodium nitroprusside (SNP) or 0.1 mM sodium cyanide (CN) was measured under hypoxic conditions induced by 95% nitrogen–5% carbon dioxide. Carboxy-PTIO (PTIO) and oxyhaemoglobin (Hb) were used at 1.0 and 0.1 mM respectively. The cell survival at 8 Gy without radiosensitiser in normoxia and hypoxia is indicated by arrows.

*Amperometric measurement of NO generated by sodium nitroprusside and S-nitroso-L-glutathione*

As NO scavengers demonstrated diminished efficiency in abrogating SNP-mediated radiosensitisation (see above), we studied in more detail the mechanism of NO release using a NO-specific microsensor. This sensor measures only free radical NO that can be directly released from NO donors in extracellular space or appears there because of diffusion from the intracellular NO pool. SNP generated NO almost exclusively by a bioreductive mechanism (Figure 7), and the low background of spontaneous NO release was probably a result of its known photosensitivity (Bates *et al.*, 1991). The addition of carboxy-PTIO resulted in complete scavenging of the NO signal in contrast to an only partial radioprotective effect (Figure 6). GSNO revealed a substantial background of spontaneous NO release and, strikingly, the total signal of extracellular NO exceeded that of SNP by 2.9-fold.

*The effect of sodium nitroprusside on radiation-induced DNA breakage*

NO is believed to radiosensitise hypoxic cells by a mechanism of DNA damage fixation similar to oxygen (Mitchell *et al.*, 1993). However, no experimental support for this hypothesis exists at present. Therefore, we examined the radiosensitising effects of SNP in relation to DNA breakage in a wide concentration range of 0.01–3 mM (Figure 8). These effects have been corrected for the direct cytotoxicity of SNP apparent at 1–3 mM. Hypoxic PSN1 cells irradiated at 8 Gy showed a reduced frequency of single-strand DNA breaks that may be attributed to the mechanism of hypoxic cell radioprotection. Hypoxic cell radiosensitisation by 0.01–0.3 mM SNP was paralleled by an increasing level of DNA breakage approaching that observed in oxic cells. The upper concentrations of SNP were cytotoxic and caused DNA damage but did not further enhance radiosensitivity or DNA breakage. It is noteworthy that the radiosensitising potency of SNP in pellets (Figure 8) and in diluted cell suspensions (Figures 3 and 6) was similar.

### Discussion

Our analysis of radiation responses in a panel of eight human pancreatic tumour cell lines suggests that both intrinsic and hypoxia-induced radioresistance may contribute to failure of local control in pancreatic cancer. To analyse cellular radioresistance we preferred to use the model-free parameter MID introduced by Fertil and Malaise (1985) rather than SF2. The enhancement ratios of radiosensitisers cannot be calculated directly from SF2 data. Furthermore, SF2 in hypoxic cells is frequently higher than 0.8 and therefore difficult to measure accurately. As determined by the MTT serial dilution assay, all cell lines possessed a high level of intrinsic radioresistance, in terms of MID values (2.4–6.5 Gy), similar to those reported by Fertil and Malaise (1985) for melanomas (2.1–2.7 Gy) and glioblastomas (1.6–4.6 Gy). This conclusion was confirmed by a colony formation assay, although the mean MID was 23% lower (1.6–4.9 Gy). Possibly, the short-term MTT assay overestimates cell survival after irradiation because non-clonogenic cells may remain metabolically active. To evaluate the radiosensitising activity of bioreductive radiosensitisers, we modified a model of metabolism-induced hypoxia previously described by Mitchell *et al.* (1993). We used a cell micropellet in plastic tips rather than a dense cell suspension as this approach requires 10–30 times fewer cells and fewer technical precautions to prevent reoxygenation. At a survival

**Figure 7** Amperometric measurement of NO generated by sodium nitroprusside and S-nitroso-L-glutathione. The release of NO from sodium nitroprusside (**a**) and S-nitroso-L-glutathione (**b**) was estimated by a Iso-NOP200 microsensor at 37°C, under a 5% nitrogen–5% carbon dioxide atmosphere. The spontaneous and bioreductive release of NO was measured respectively in the absence (○) and presence (△) of PSN1/ADR cells ($32 \times 10^6$ cells ml$^{-1}$). The NO scavenger carboxy-PTIO (PTIO) was added after 6 min incubation (closed symbols) to produce a final concentration of 1 mM.

**Figure 8** Concentration-dependent effects of sodium nitroprusside on radiosensitivity (**a**) and DNA breakage (**b**) in hypoxic PSN1 cells irradiated at 8 Gy. The cell survival fraction (○, ●) and the frequency of single-strand DNA breaks (△, ▲) were measured for cells exposed to sodium nitroprusside alone (open symbols) or with radiation (closed symbols). The dashed area indicates the sensitising effect of sodium nitroprusside. The cell survival and DNA breakage at 8 Gy without radiosensitiser in normoxia and hypoxia are indicated by arrows.

fraction of 0.1, OER was 2.7–2.9, indicating radiobiologically relevant hypoxia, as also observed in the model of Mitchell *et al.* (1993).

SNP, a well-known bioreductive NO donor (Bates *et al.*, 1991), has been found to be an efficient radiosensitiser of hypoxic cells at non-cytotoxic concentrations between 0.01 and 0.3 mM. In a panel of eight lines, SNP at 0.1 mM showed a mean enhancement ratio of 1.9 compared with 2.5 for oxygen. The molar radiosensitising potency of SNP was at least 10-fold higher than that of etanidazole, a representative of 2-nitroimidazoles undergoing clinical trials. Interestingly, at survival fractions above 0.3, which are attributed to clinically relevant doses of radiation (2–4 Gy), SNP demonstrated an increased radiosensitising effect, whereas oxygen showed a reduced effect. This phenomenon resembles the recent findings on etanidazole and buthionine sulphoximine, whose radiosensitising effects, in contrast to oxygen, were increased at high survival fractions (Skov and MacPhail, 1992, 1994). Under aerobic conditions SNP was not active, presumably because of the rapid oxidation of NO. This suggests that SNP is a specific radiosensitiser of hypoxic cells, and that the same target could be implicated in the mechanisms of SNP and oxygen-mediated radiosensitisation. This point of view is supported by our data on radiation-induced DNA breakage under hypoxic conditions. SNP in the range of 0.01–0.3 mM caused a concentration-dependent enhancement of DNA breakage up to the level observed in the presence of oxygen.

The mechanism of SNP-mediated radiosensitisation appears to be complex as this agent generates two radiobiologically active products – NO and CN. Douple and Green (1980) described radiosensitisation of hypoxic and oxic cells by SNP after a prolonged exposure time of 2 h, and speculated that SNP-derived CN may inhibit cellular respiration and thereby 'spare' oxygen, a natural radiosensitiser. However, no evidence of the radiosensitising activity of CN itself was provided, and the accumulation rate of CN in monolayer cultures was not estimated by the authors. Our short-term experiments in cell suspensions with controlled rates of SNP decomposition suggest that even complete liberation of CN from SNP could account for less than 17% of the radiosensitising effect. Instead, the role of NO is supported by the reversing effect of the NO scavengers carboxy-PTIO and oxyhaemoglobin on the radiosensitising action of SNP and GSNO. Moreover, NO in gaseous form or released from DEA/NO has previously been shown to radiosensitise hypoxic cells (Mitchell *et al.*, 1993). Therefore, we conclude that SNP-derived NO by itself can cause hypoxic cell radiosensitisation. On the other hand, CN may contribute to the radiosensitising effect of SNP in the *in situ* models of metabolic hypoxia as extensively discussed by Douple and Green (1981). As the role of CN in SNP-induced radiosensitisation appears to be limited, we focused further analysis on the NO-mediated mechanism. We considered NO as the common mediator responsible for the radiosensitising effects of GSNO and SNP. GSNO was involved in our study as a pure NO releaser, and has been implicated in the intracellular pathways of NO redox forms (Ignarro *et al.*, 1981; Stamler *et al.*, 1992; Clancy *et al.*, 1994).

The bioreductive generation of NO from SNP is thought to proceed via a one-electron transfer reduction at the cellular membrane, which operates as a catalyser (Bates *et al.*, 1991; Rochelle *et al.*, 1994). Thus, the half-life of SNP should be correlated inversely with cell density, consistent with our observations. In the case of pellets, the calculated half-life was around 1 min, indicating complete decomposition of SNP at the time of irradiation. However, we found approximately the same radiosensitising activity in pellets as in diluted cell suspensions, wherein the rate of SNP bioactivation declined by a factor of 30–100. Therefore, the rate of NO generation is not predictive for the radiosensitising activity of SNP. We hypothesise that in hypoxic cells an effective accumulation of SNP-derived NO may occur at any cell density by transfer of the NO radical to cellular targets, without its liberation into the medium. This is in keeping with the reduced potency of the NO scavengers carboxy-PTIO and oxyhaemoglobin to counteract the radiosensitising effect of SNP compared with that of GSNO. The model of a direct transfer of the SNP-derived nitrosonium cation on membrane-bound thiols has already been suggested (Rochelle *et al.*, 1994). Direct measurements of the extracellular NO concentration, using a specific microsensor, are consistent with the above model. Firstly, we detected only marginal spontaneous liberation of NO from SNP in the absence of cells. Secondly, bioreductive activation of SNP did not result in the generation of free NO in the extracellular space at a rate that could explain the 10-fold higher radiosensitising potency of SNP compared with that of GSNO. Finally, the radiosensitising effect of SNP was only partially abrogated by carboxy-PTIO, although the extracellular NO signal completely disappeared. We stress again that in cell suspensions the radiosensitising effect of CN was negligible and could not explain the discrepancy between the radioprotective and scavenging properties of carboxy-PTIO. Therefore, intracellular NO adducts, but not the extracellular pool of free NO, may confer SNP-mediated radiosensitisation.

In contrast to SNP, GSNO spontaneously liberated NO into the medium and generated higher extracellular concentrations of NO by bioreduction. This increased pool of extracellular NO was contradictory to the lower radiosensitising activity, which again confirms the lack of correlation between NO production and radiosensitising activity for bioreductive NO donors. Endogenously formed GSNO is conceivably the major adduct of natural thiols, with nitrosonium cation accumulated in cells after exposure to NO (Ignarro *et al.*, 1981; Stamler *et al.*, 1992; Clancy *et al.*, 1994). As such, it may also be involved in the trapping and storing of SNP-derived NO adducts in hypoxic cells, thereby supporting delayed radiosensitisation. On the other hand, increased levels of intracellular glutathione may slow down the transfer of the SNP-derived NO towards DNA as a result of trapping of the nitrosonium cation, and sustain intrinsic radioresistance at the same time (Skov and MacPhail, 1992). This could be one of the mechanisms underlying a decreased radiosensitising effect of SNP in the more radioresistant cell lines A818-7, Colo-357, Panc-1 and HPAF. Hence, the radiosensitising potency of SNP can be reduced in slow-growing and well-differentiated tumour cells possessing elevated radioresistance.

Recently, the NONOate complex DEA/NO has been proposed as a hypoxic tumour cell radiosensitiser with a dual action on both tumour and vascular cells (Mitchell *et al.*, 1993). The authors speculated that, besides direct sensitisation of hypoxic tumour cells, DEA/NO may improve tumour oxygenation and radiosensitisation because of the vasodilatory effect on tumour vasculature. However, the reduction in tumour blood flow and oxygenation due to preferential peripheral vasodilatation and decrease in blood pressure is also possible. Indeed, these alternative effects have been already described for the vasodilators flunarizine and hydralazine (discussed in Teicher *et al.*, 1993). Another limitation to the use of NO prodrugs may arise from their side-effects on the nervous systems, which is responsive to low concentrations of NO (Knowles and Moncada, 1994). Therefore, the concentrations of NO required for radiosensitisation may be difficult to obtain *in vivo* for any NO donor. The minimum concentration of SNP expected to exert radiosensitisation is close to 0.01 mM. Such a concentration in plasma is achievable at subtoxic doses in mice (Smith and Kruszyna, 1974), but in humans a safety infusion protocol of SNP used to maintain vasodilatation ($<3\ \mu g\ min^{-1}\ kg^{-1}$) would not result in a sufficient level of NO equivalents. The same problem applies to DEA/NO, which spontaneously liberates NO with a constant half-life of 2.1 min (Maragos *et al.*, 1993). In metabolically induced hypoxia, DEA/NO showed half-reversal of radioprotection at 0.3–0.5 mM, whereas SNP reveals the same effect at a concentration of 0.1 mM and at similar or longer half-lives. The comparison of the SNP and DEA/NO potency indicates that bioreductive NO donors may evoke radiosensitising action at reduced pharmacokinetic doses, a feature claimed also for other bioreductive modifiers that exploit redox conditions in hypoxic cells (Adams, 1992). At present, it is not clear whether redox microenvironments in tumours might favour the accumulation of NO intermediates up to radiobiologically active levels at tolerated doses of SNP. However, the radiosensitising effect of infused SNP combined with carbogen breathing has been clearly observed in Lewis carcinoma-bearing mice (Teicher *et al.*, 1993), thus warranting further *in vivo* studies of this promising bioreductive radiosensitiser.

In conclusion, our studies in human pancreatic tumour cell lines have provided a radiobiological rationale for the poor radioresponsiveness in pancreatic cancer based on high intrinsic cellular radioresistance. Additionally, we demonstrated that SNP, a bioreductive NO donor, elicits efficient hypoxic cell radiosensitisation through the generation of NO and fixation of DNA damage. SNP, an active vasodilatory drug with a long history in the treatment of hypertension, may also be of value as a tumour radioresponse modifier.

### Abbreviations

SNP, sodium nitroprusside; GSNO, S-nitroso-L-glutathione; NO, nitric oxide; DEA/NO, complex of diethylamine with NO; MID, mean inactivation dose; SF2, survival fraction at 2 Gy; OER, oxygen enhancement ratio.

### Acknowledgements

This research was funded by grants nos. 3.0036.94 and G.0064.95 from the National Fonds voor Wetenschappelijk Onderzoek (NFWO) and Sportvereniging tegen Kanker. The authors wish to thank Mrs C Monsaert for valuable technical support. We are grateful to Prof Dr M Mareel for critical review. We also acknowledge Prof Dr G Klöppel, who kindly provided the human pancreatic tumour cell lines.

### References

ADAMS GE. (1992). Redox, radiation and reductive bioactivation. *Radiat. Res.*, **132**, 129–139.

AKAIKE T, YOSHIDA M, MIYAMOTO Y, SATO K, KOHNO M, SASAMOTO K, MIYAZAKI K, UEDA S AND MAEDA H. (1993). Antagonistic action of imidazolineoxyl N-oxides against endothelium-derived relaxing factor/•NO through a radical reaction. *Biochemistry*, **32**, 827–832.

BATES JN, BAKER MT, GUERRA R AND HARRISON DG. (1991). Nitric oxide generation from nitroprusside by vascular tissue: evidence that reduction of the nitroprusside anion and cyanide loss are required. *Biochem. Pharmacol.*, **42**, S157–S165.

BRENNAN MF, TIMOTHY JK AND CASPER ES. (1993). Cancer of the pancreas. In *Cancer: Principles & Practice of Oncology, 4th edition*, DeVita Jr VT, Hellman S and Rosenberg SA (eds) pp 849–820. Lippincott: Philadelphia.

CARMICHAEL J, DEGRAFF WG, GAZDAR AF, MINNA JD AND MITCHELL JB. (1987). Evaluation of a tetrazolium-based semiautomated colorimetric assay: assessment of radiosensitivity. *Cancer Res.*, **47**, 943–946.

CLANCY RM, LEVARTOVSKY D, LESZCZYNSKA-PIZIAK J, YEGUDIN J AND ABRAMSON SB. (1994). Nitric oxide reacts with intracellular glutathione and activates the hexose monophosphate shunt in human neutrophils: evidence for S-nitrosoglutathione as a bioactive intermediary. *Proc. Natl Acad. Sci. USA*, **91**, 3680–3684.

DELVAEYE M, VEROVSKI V, DE NEVE W AND STORME G. (1993). DNA breakage, cytotoxicity, drug accumulation and retention in two human ovarian tumor cell lines AZ224 and AZ364 treated with adriamycin, modulated by verapamil. *Anticancer Res.*, **13**, 1533–1538.

DOUPLE EB AND GREEN CJ. (1980). Potentiation of cellular radiosensitivity by nitroprusside and vitamin $B_{12}$. *Int. J. Radiat. Oncol. Biol. Phys.*, **6**, 1545–1549.

FERTIL B AND MALAISE EP. (1985). Intrinsic radiosensitivity of human cell lines is correlated with radioresponsiveness of human tumors: analysis of 101 published survival curves. *Int. J. Radiat. Oncol. Biol. Phys.*, **11**, 1699–1707.

HOWARD-FLANDERS P. (1957). Effect of nitric oxide on the radiosensitivity of bacteria. *Nature*, **180**, 1191–1192.

IGNARRO LJ, LIPPTON H, EDWARDS JC, BARICOS WH, HYMAN AL, KADOWITZ PJ AND GRUETTER CA. (1981). Mechanism of vascular smooth muscle relaxation by organic nitrates, nitrites, nitroprusside and nitric oxide: Evidence for the involvement of S-nitrosothiols as active intermediates. *J. Pharmacol. Exp. Ther.*, **218**, 739–749.

KNOWLES RG AND MONCADA S. (1994). Nitric oxide synthases in mammals. *Biochem. J.*, **298**, 249–258.

MAILLET B, DE GREVE J, LEMOINE N, KALTHOFF H, SCHMIEGEL W AND KLOPPEL G. (1993). Phenotypical differentiation and genetic alterations in human pancreatic carcinoma cell lines. *Int. J. Pancreatol.*, **14**, 72-75.

MARAGOS CM, MORLEY D, WINK DA, DUNAMS TM, SAAVEDRA JE, HOFFMAN A, BOVE AA, ISAAC L, HRABIE JA AND KEEFER LK. (1991). Complexes of •NO with nucleophiles as agents for the controlled biological release of nitric oxide. Vasorelaxant effects. *J. Med. Chem.*, **34**, 3242-3247.

MARAGOS CM, WANG JM, HRABIE JA, OPPENHEIM JJ AND KEEFER LK. (1993). Nitric oxide/nucleophile complexes inhibit the in vitro proliferation of A375 melanoma cells via nitric oxide release. *Cancer Res.*, **53**, 564-568.

MITCHELL JB, WINK DA, DEGRAFF W, GAMSON J, KEEFER LK AND KRISHNA MC. (1993). Hypoxic mammalian cell radiosensitization by nitric oxide. *Cancer Res.*, **53**, 5845-5848.

ROCHELLE LG, KRUSZYNA H, KRUSZYNA R, BARCHOWSKY A, WILCOX DE AND SMITH RP. (1994). Bioactivation of nitroprusside by porcine endothelial cells. *Toxicol. Appl. Pharmacol.*, **128**, 123-128.

SKOV KA AND MACPHAIL HS. (1992). Effect of BSO on the radiation response at low (0-4 Gy) doses. *Int. J. Radiat. Oncol. Biol. Phys.*, **22**, 533-536.

SKOV KA AND MACPHAIL HS. (1994). Low concentrations of nitroimidazoles: effective radiosensitizers at low doses. *Int. J. Radiat. Oncol. Biol. Phys.*, **29**, 87-93.

SMITH RP AND KRUSZYNA H. (1974). Nitroprusside produces cyanide poisoning via a reaction with hemoglobin. *J. Pharmacol. Exp. Ther.*, **191**, 557-563.

STAMLER JS, SINGEL DJ AND LOSCALZO J. (1992). Biochemistry of nitric oxide and its redox-activated forms. *Science*, **258**, 1898-1902.

TEICHER BA, HOLDEN SA, NORTHEY D, DEWHIRST MW AND HERMAN TS. (1993). Therapeutic effect of infused fluosol-DA/carbogen with ephedrine, flunarizine, or nitroprusside. *Int. J. Radiat. Oncol. Biol. Phys.*, **26**, 103-109.

VEROVSKI VN, VAN DEN BERGE DL, DELVAEYE MM, SCHEPER RJ, DE NEVE WJ AND STORME GA. (1996). Low-level doxorubicin resistance in P-glycoprotein-negative human pancreatic tumour PSN1/ADR cells implicates a brefeldin A-sensitive mechanism of drug extrusion. *Br. J. Cancer*, **73**, 596-602.